# Resisting infection by *Plasmodium berghei* increases the sensitivity of the malaria vector *Anopheles gambiae* to DDT

**DOI:** 10.1186/s12936-015-0646-y

**Published:** 2015-03-28

**Authors:** Adam Saddler, Paul-Christian Burda, Jacob C Koella

**Affiliations:** Division of Biology, Faculty of Life Sciences, Imperial College London, Silwood Park Campus, Ascot, Berkshire SL5 2PZ UK; Faculté des Sciences, Institut de Biologie, Université de Neuchâtel, Rue Emile-Argand 11, CH-2000 Neuchâtel, Switzerland; Department of Health Interventions, Swiss Tropical and Public Health Institute, Socinstrasse, 57, CH-4002 Basel, Switzerland; Ifakara Health Institute, Box 74, Bagamoyo, Tanzania; University of Basel, Petersplatz 1, Basel, 4003 Switzerland; Institute of Cell Biology, University of Bern, Bern, Switzerland

**Keywords:** Insecticide, Resistance phenotypes, *Anopheles gambiae*, *Plasmodium berghei*

## Abstract

**Background:**

The evolution of insecticide resistance threatens current malaria control methods, which rely heavily on chemical insecticides. The magnitude of the threat will be determined by the phenotypic expression of resistance in those mosquitoes that can transmit malaria. These differ from the majority of the mosquito population in two main ways; they carry sporozoites (the infectious stage of the *Plasmodium* parasite) and they are relatively old, as they need to survive the development period of the malaria parasite. This study examines the effects of infection by *Plasmodium berghei* and of mosquito age on the sensitivity to DDT in a DDT-resistant strain of *Anopheles gambiae*.

**Methods:**

DDT-resistant *Anopheles gambiae* (ZANU) mosquitoes received a blood meal from either a mouse infected with *Plasmodium berghei* or an uninfected mouse. 10 and 19 days post blood meal the mosquitoes were exposed to 2%, 1% or 0% DDT using WHO test kits. 24 hrs after exposure, mortality and *Plasmodium* infection status of the mosquitoes were recorded.

**Results:**

Sensitivity to DDT increased with the mosquitoes’ age and was higher in mosquitoes that had fed on *Plasmodium*-infected mice than in those that had not been exposed to the parasite. The latter effect was mainly due to the high sensitivity of mosquitoes that had fed on an infected mouse but were not themselves infected, while the sensitivity to DDT was only slightly higher in mosquitoes infected by *Plasmodium* than in those that had fed on an uninfected mouse.

**Conclusions:**

The observed pattern indicates a cost of parasite-resistance. It suggests that, in addition to the detrimental effect of insecticide-resistance on control, the continued use of insecticides in a population of insecticide-resistant mosquitoes could select mosquitoes to be more susceptible to *Plasmodium* infection, thus further decreasing the efficacy of the control.

## Background

Mosquito control with insecticides has been an important tool in the fight against malaria since Paul Müller discovered the insecticide properties of DDT in the late 1930s [1]. Bed nets treated with insecticides and indoor residual spraying of insecticide are now the mainstay of malaria control. However, the evolution of insecticide resistance in mosquitoes threatens the success of these techniques. Resistance to each chemical class of insecticide used for malaria control has now been recorded, and resistance can be found in many malarious regions [2-4]. Understanding the evolution of resistance is therefore crucial to maintain effective programmes of malaria control. To aid this understanding test protocols have been developed, enabling easy identification and monitoring of resistant mosquitoes in the field [5]. Furthermore, many of the molecular mechanisms and genetic factors underlying insecticide-resistance have been identified [6]. It is, however, the phenotypic expression of resistance that threatens malaria control, so it is crucial that any environmental or demographic effects interacting with the genetic basis of resistance are understood.

Environmental stresses such as temperature [7-9], food source [10,11] and the availability of blood-meals [12] can influence the expression of resistance. Infection by fungal [13] and microsporidian [14] parasites can also increase sensitivity to insecticide. The mechanisms behind the increase in sensitivity to parasitism are unknown, but may include increased metabolic stress caused by the pathogens and the re-allocation of detoxification enzymes from defence against the insecticide towards defence against the parasite [13,14]. It is therefore possible that natural parasites of mosquitoes may also have an impact on the expression of resistance; the most important for malaria control being the malaria parasite itself.

Whether infection by *Plasmodium* affects the expression of resistance has been investigated in two studies, with contradictory results. In one, infection by *Plasmodium yoelii* or *Plasmodium chabaudi* did not change the sensitivity of permethrin-sensitive *Anopheles stephensi* mosquitoes to low doses of permethrin [15]. In contrast, in resistant *Anopheles gambiae* (homozygous for the *kdr* mutation) infection by *Plasmodium falciparum* increased the mortality of mosquitoes after exposure to DDT [16].

The latter study confirms that the sensitivity to insecticides increases with the mosquito’s age, [17-24]. It also suggests that in older mosquitoes, that are potentially harbouring sporozoite-stage parasites, the effect of the parasite on resistance decreases (though different analyses gave conflicting results).

The following study investigates the impact of infection by the malaria parasite *Plasmodium berghei* and of the mosquito’s age on the phenotypic expression of resistance in mosquitoes that are genetically resistant to the insecticide dichlorodiphenyltrichloroethane (DDT). The study adds to previous work by (i) comparing the mosquitoes that are infected by malaria and those that have fed on malaria-infected blood but are not themselves infected, and (ii) comparing different doses of the insecticide.

## Methods

### Experimental design

Experiments were carried out using a DDT-resistant colony of the mosquito *An. gambiae* (ZANU), which has increased metabolism of the insecticide, catalyzed by members of the glutathione S-transferase enzyme family [25]. Infected and uninfected mosquitoes were exposed to filter paper treated with, 2% DDT, 1% DDT or 0% DDT (paper containing oil as a control) for 1 hr using the standard WHO test kits [26]. Insecticide exposures on mosquitoes were carried out 10 or 19 days after blood feeding in order to capture two stages of the malaria parasite, non-transmissible oocysts (day 10) and infectious sporozoites (day 19). Mosquito survival was recorded 24 hours after exposure.

4% DDT is the standard dose used to discriminate resistant mosquitoes from sensitive mosquitoes [26]. However, resistance decreases strongly with age in the ZANU mosquitoes [21], leading to close to 100% mortality in old mosquitoes. To ensure sufficient variability in the data, the lower doses were used to test the effects of infection and age on sensitivity.

### Mosquito rearing

Mosquitoes were reared at a temperature of 26 (+/-2)°C and 70 (+/-10) % relative humidity with a 12 h:12 h light/dark cycle. 1500 larvae were reared individually in 12-well plates and fed Tetramin fish food (0.04 mg at age 1 day, 0.08 mg at age 2, 0.16 mg at age 3, 0.32 mg at age 4, 0.6 mg at ages 5 and older) until pupation.

Pupae were transferred to cages (20 cm × 20 cm × 20 cm). The mosquitoes remained in the emergence cages for 48 hrs to give them time to mate. Then female mosquitoes were separated into 8 cages. Adults were supplied with 10% glucose solution, which was removed 24 hrs before blood feeding on mice when they were 4 or 5 days old.

### Blood feeding and infection with *Plasmodium berghei*

24 hrs before blood feeding mosquitoes were transferred to a climate-chamber with the temperature of 19 (+/-2)°C, which is the optimal temperature for *P. berghei* development [27]. Mosquitoes remained in this chamber until the completion of the experiment, including during DDT exposures.

Four Balb/c mice infected with *P. berghei,* expressing Green Fluorescent Protein (PbGFPCON strain, [28]), and four uninfected mice that were, apart from the infection, treated identically were obtained from the Heussler research group, University of Bern, Switzerland. One mouse was assigned haphazardously to each cage and the mosquitoes were allowed to feed for 45 minutes.

24 hours after feeding, the mosquitoes that had taken a blood meal were divided into six cups per cage (one cup per dose and timing of insecticide exposure) and supplied daily with fresh sugar water until they were exposed to the insecticide.

After recording survival (24 hours after the insecticide exposure) the mosquitoes were checked for infection by *Plasmodium* by dissecting the mosquitoes’ midguts exposed to *Plasmodium* and counting the oocysts at 100x magnification using a fluorescent microscope. For the mosquitoes exposed to DDT 19 days after the blood meal, the salivary glands were also checked for the presence of sporozoites.

### Statistical analysis

The analyses were carried out with R version 3.0.3. The first analysis considered the effect of exposure to the malaria parasite, i.e. the effect of having fed on an infected mouse. Mosquito survival 24 hrs after exposure was analysed with a binomial generalized linear model (GLM) with logit link, corrected for possible over- or underdispersion. Day of exposure, concentration of DDT, exposure to *Plasmodium* and all interactions were included as fixed, nominal factors, and the mouse a mosquito had fed on was included as a random factor that is nested within exposure.

No *Plasmodium* parasites were found in 31% of the exposed mosquitoes, therefore a second analysis was conducted where the effect of infection was considered. The data were grouped into three categories: mosquitoes that had fed on an uninfected mouse, mosquitoes that had fed on an infected mouse but were not infected themselves and mosquitoes that had fed on infected mice and were infected. The mice were not included, as the first analysis showed that they explained less than 1% if the variance and because the design did not allow them to be nested within infection.

## Results

The mouse a mosquito had fed on had no effect on the sensitivity to the insecticide, explaining less than 1% of the variance in mortality 24 hours after exposure to the insecticide.

Mosquitoes were more likely to feed on uninfected mice (30 mosquitoes per treatment group) than on infected mice (23 mosquitoes per treatment group). Oocysts we found in 52 out of 69 mosquitoes (on day 10) that had fed on infected mice and sporozoites in 41 out of 66 mosquitoes (on day 19). The difference in infection between days was not significant, (χ^2^ = 2.77, p = 0.1).

24-hour mortality increased from three out the 102 mosquitoes that were not exposed to DDT to 89 of the 98 exposed to the highest dose. Mortality was higher on day 19 (69 out of 154) than on day 10 (53 out of 161). Both effects were highly significant (Table 1).

**Table 1:** **Analysis where the effect of** ***Plasmodium*** **was considered as feeding on** ***Plasmodium*** **-infected blood**

**Factor**	**Df**	**Deviance**	**Residual df**	**Residual deviance**	**p**
*Plasmodium*-fed	1	2.15	46	255.6	0.064
Dose of DDT	2	201.37	44	54.3	<0.001
Age at challenge by DDT	1	13.68	43	40.6	<0.001
*Plasmodium*-fed* dose	2	7.96	41	32.7	0.002
*Plasmodium*-fed* age	1	0.39	40	32.3	0.429
Dose* age	2	1.50	38	30.8	0.302
*Plasmodium*-fed* dose * age	2	6.37	36	24.4	0.006

*notes interaction term.

**Figure 1:**
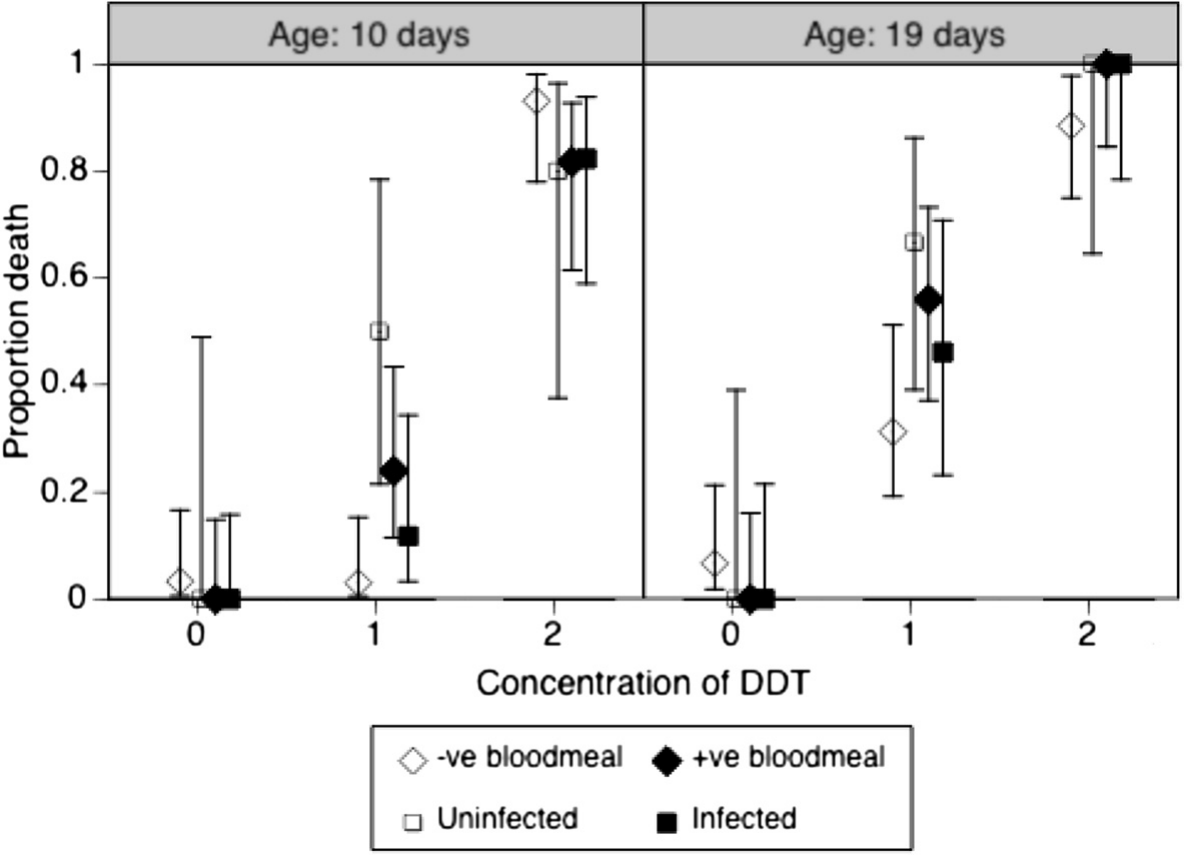
**Proportion of mosquitoes dying within 24 hours after exposure to 0%, 1% or 2% DDT.** Exposures occurred on 10 days (left) or 19 days (right) after blood feeding on mice infected with *Plasmodium*. The diamonds represent mosquitoes that had fed on an uninfected mouse (open diamonds) and those fed on an infected mouse (solid diamonds). The squares consider only the mosquitoes fed on an infected mouse, and represent those in which we found parasites (solid squares) and those that had no parasites (open squares). The vertical lines show the 95% confidence intervals of the proportions.

Feeding on a *Plasmodium*-infected mouse increased the mortality from 36% to 44%, though this difference was not quite statistically significant (p = 0.06, Table 1). The effect depended strongly on the dose of DDT. With no exposure, mortality was very low (<5%) among mosquitoes that had fed on infected or on uninfected mice; at the highest dose mortality was high (>90%) irrespective of *Plasmodium*-status (Figure 1). However, at the intermediate dose of DDT mosquitoes that had fed on uninfected mice were only slightly affected (17% mortality), whereas 40% of the mosquitoes that had fed on an infected mouse were killed. Although the details of this interaction between exposure to *Plasmodium* and exposure to DDT depend on the age of the mosquito (Table 1), the pattern is similar at the two ages (Figure 1).

A similar pattern was observed in the second analysis where the mosquitoes were analysed according to their infection (Figure 1). In particular, the mortality after exposure to DDT depended on the interaction between *Plasmodium* and the dose of DDT, and this interaction was influenced by age (Table 2). The main result of this analysis was that the mortality of infected mosquitoes was very close to that of control mosquitoes, and that the effect of exposure to *Plasmodium* on DDT-induced mortality was most apparent for mosquitoes that had fed on an infected mouse, but were themselves not infected (Figure 1).

**Table 2:** **Analysis according to infection status**

**Factor**	**Df**	**Deviance**	**Residual df**	**Residual deviance**	**p**
Infection by *Plasmodium*	2	5.17	64	266.9	0.014
Dose of DDT	2	204.14	62	62.8	<0.001
Age at challenge by DDT	1	12.36	61	50.4	<0.001
Infection* dose	4	7.90	57	42.5	0.011
Infection* age	2	1.16	55	41.3	0.380
Dose* age	2	1.64	53	39.7	0.256
Infection* dose* age	4	6.28	49	33.4	0.034

For infection status three categories were considered: mosquitoes that had fed on uninfected blood, mosquitoes that were infected, and mosquitoes that had fed on infected blood but did not harbour any parasites. *notes interaction term.

## Discussion

This study, with *P. berghei* and *An. gambiae,* found, (i) that sensitivity to insecticide increased with age and exposure to *Plasmodium* parasites, (ii) that infection by the *Plasmodium* parasite had no effect on the expression of insecticide resistance, while the mosquitoes that had fed on *Plasmodium*-infected blood but were not themselves infected were more sensitive than either infected or control mosquitoes and (iii) that *Plasmodium* affected the expression of insecticide similarly in young and old mosquitoes.

That sensitivity increases with age of the mosquito is no surprise, and has been shown in several species of mosquito, in mosquitoes with metabolic resistance and in mosquitoes with target site resistance [18-24].

The effect of *Plasmodium* on sensitivity corroborates one study [15], in which infection by *P. yoelii* or *P. chabaudi* had no effect on sensitivity. However, it contrasts a more recent study on *P. falciparum* [16], where infection increased sensitivity. Several experimental differences may be responsible for the variability of the results: the use of different mosquito and parasite species, the use of different mechanisms of insecticide resistance, the use of different types and doses of insecticides. Whatever the reason, it is clear that more information about the role of such differences is needed before definitive statements about the effect of *Plasmodium* infection on the expression of insecticide-resistance can be made.

Finally, while the quantitative details of the interaction between *Plasmodium* and exposure to the insecticide changed with age (Figure 1), the overall pattern is similar in young and old mosquitoes: the sensitivity of infected mosquitoes was similar to that of controls, while that of exposed but uninfected mosquitoes was higher. This consistency across ages again contrasts with the age-specific pattern observed by Alout *et al.* [16]. The simplest explanation is that they used a dose of insecticide that killed most mosquitoes when they were old, masking any possible effect of the infection.

That exposed mosquitoes affected the expression of resistance only if they were not infected suggests a trade-off between mounting an effective immune response and surviving DDT exposure. The mosquito’s immune response against *Plasmodium* is mediated partly by the expression of detoxification enzymes, particularly cytochrome P450 and the glutathione-S-transferases [29,30]. Such detoxification enzymes are also associated with metabolic resistance against insecticides, which is the main mechanism for the ZAN/U strain used in the current study. Diverting these enzymes to the defence against malaria may leave the resistant mosquitoes more vulnerable to insecticide exposure [31].

In addition, immune responses can be energetically costly [32-34], leaving fewer energy reserves to fight the insecticide. Indeed, this cost is expected to be especially high for insecticide-resistant mosquitoes, for resistant mosquitoes store fewer lipids, sugars and energetic reserves than sensitive ones [35], making the cost of the immune response more apparent. Thus, it is hypothesized that the change in expression of detoxification genes induced by infection and the energetic cost of the immune response would lead to a trade-off between the elimination of the parasite and surviving the insecticide.

The most worrying aspect of the study is that it suggests that the ability to clear parasites is associated with lower resistance to insecticides. If this is common in natural populations (although one study suggests that it may not be [16]), continued use of insecticides in populations of insecticide-resistant populations would lead to the death of mostly parasite-resistant mosquitoes, thus increasing the potential for transmission, further enhancing the susceptibility of mosquitoes that evolved as a correlated response to the evolution of insecticide-resistance [36].

The encouraging aspect of the study is that no mosquitoes that fed on infected mice, whether they were subsequently infected or not, survived an exposure to 2% DDT 19 days after the blood meal. The genetically resistant ZANU mosquitoes would therefore be classed as sensitive by the time they can transmit the parasite (according WHO recommendations of 4% DDT as the discriminating dose [26]). If this pattern is similar natural populations, resistant mosquitoes would pose less of a threat than is traditionally believed. However, the resistance of ZANU mosquitoes drops more dramatically with age than what has been observed in other studies [18-20,22-24].

Thus the impact of insecticide-resistance on malaria control may be influenced by the strengths of two opposing forces: (i) decreasing resistance with age, which decreases transmission, and (ii) selection against *Plasmodium* resistant mosquitoes, which increases transmission. This underscores the complexity of ecological factors, such as the costs of insecticide-resistance on the mosquitoes’ survival and fecundity, that determine the degree to which insecticide resistance will impact malaria control [37].

Before reaching strong conclusions, it must be understood why different studies with, among others, different mosquito and parasite species and with different insecticide-resistance mechanisms, give contrasting results. Nevertheless, the results here suggest that resistance to *Plasmodium* is traded-off with resistance to insecticides, which would have important consequences for the control of malaria by insecticides.
